# Meta-analysis of the effect of self-management behavior intervention measures in older adults with coronary heart disease and diabetes mellitus

**DOI:** 10.3389/fcvm.2026.1821784

**Published:** 2026-08-12

**Authors:** Jing Yang, Peiling Zhang, Rui Yang, Xu Yuan, Xian Li, Bei Wang, Jingjing Wang

**Affiliations:** 1 Cardiovascular Surgery, Second Hospital of the Naval Medical University, Shanghai, China; 2 Nursing Department, Second Hospital of the Naval Medical University, Shanghai, China

**Keywords:** coronary heart disease, diabetes mellitus, meta-analysis, older adults, self-management behavior

## Abstract

**Background:**

With population aging, the prevalence of older adults with coronary heart disease (CHD) and diabetes mellitus (DM) is increasing. These patients face complex disease burdens, and self-management behaviors are crucial for disease control and prognosis. However, current interventions show inconsistent results. This meta-analysis systematically evaluates the effects of self-management behavior interventions in older adults with CHD and DM.

**Methods:**

PubMed, Web of Science, and Embase were searched from inception to February 2025 for randomized controlled trials on self-management behavior interventions in older adults with CHD and DM. Two researchers independently screened studies, extracted data, and assessed bias risk. Nine studies involving 1757 patients were included. The primary outcome was the self-management behavior score; secondary outcomes were HbA1c, fasting plasma glucose (FPG), total cholesterol (TC), and cardiovascular adverse event incidence. Meta-regression and sensitivity analyses (leave-one-out method) were conducted to explore sources of heterogeneity. The GRADE approach was used to assess evidence certainty.

**Results:**

Compared with conventional care, comprehensive self-management behavior interventions significantly improved self-management scores [SMD = 1.32, 95% CI (1.10–1.54), *P* < 0.05]. Biochemical markers significantly improved in intervention groups: HbA1c [SMD = −0.91, 95% CI (−1.10 to −0.72)], FPG [SMD = −0.87, 95% CI (−1.06 to −0.68)], and TC [SMD = −0.80, 95% CI (−0.98 to −0.62)], all *P* < 0.05. Cardiovascular adverse event incidence was significantly lower in intervention groups [RR = 1.77, 95% CI (1.73–1.82), *P* < 0.05]. Substantial statistical heterogeneity was observed for the primary outcome (*I*^2^ = 79.2%). Meta-regression identified intervention type (digital vs. non-digital) as a significant moderator [coefficient = 0.52, 95% CI (0.18–0.86), *P* = 0.008], explaining 34.2% of between-study variance. Sensitivity analysis confirmed that no single study drove the pooled effect, supporting the robustness of the result.

**Conclusion:**

Comprehensive self-management behavior interventions effectively enhance self-management levels, improve glucose and lipid metabolism, and reduce cardiovascular events in older adults with CHD and DM. However, the substantial heterogeneity (*I*^2^ = 79.2%) indicates that pooled effect sizes should be interpreted with caution; intervention type (digital vs. non-digital) is a significant contributor to heterogeneity. Future high-quality RCTs with standardized outcome measures are needed to refine these estimates.

**Systematic Review Registration**: https://www.crd.york.ac.uk/PROSPERO/, identifier CRD42025271896.

## Introduction

With global population aging, prevention and management of age-related diseases have become a public health priority. Coronary heart disease (CHD) and diabetes mellitus (DM), both highly prevalent among older adults, frequently coexist and interact, complicating treatment and worsening quality of life and prognosis (1, 2). CHD results from coronary atherosclerosis, causing myocardial ischemia, hypoxia, or necrosis (3), whereas DM is defined by chronic hyperglycemia that induces cardiovascular complications (4). In older patients, hyperglycemia accelerates atherosclerotic progression, while myocardial ischemia disrupts glucose metabolism, creating a vicious cycle that advances both conditions (5, 6). Self-management behavior plays a key role in the disease control of older adults with coronary heart disease and diabetes. Self-management means that individuals manage and control their own diseases through active monitoring of disease conditions, reasonable diet, appropriate exercise, correct medication, and regular medical treatment (7). Good self-management behavior can effectively control blood glucose and lipid levels, reduce the risk of cardiovascular events, and thus improve the prognosis of patients (8). However, older adults with coronary heart disease combined with diabetes often have many problems in their self-management behavior due to physical function decline, cognitive function decline, and serious disease burden (9). Some older adults may have difficulty understanding complex disease management knowledge and cannot accurately monitor blood sugar, blood pressure, and other indicators; Some patients also have difficulty adhering to regular diet and exercise plans due to physical discomfort or lack of family support, resulting in poor self-management (10).

At present, there are many kinds of self-management behavioral interventions for older adults with coronary heart disease combined with diabetes, but the effects are uneven (11–13). Some studies try to improve patients' self-management ability through traditional ways, such as health education lectures and personalized diet and exercise guidance, but in practical application, it is found that these methods have limited sustainability and effectiveness in changing patients' behavior (14–16). With the development of information technology, intervention methods based on emerging technologies such as SMS and mobile applications have gradually emerged (17, 18). These emerging interventions have the characteristics of convenience, efficiency, and personalization, and can provide real-time disease management support for patients. However, in practical application, they also face problems such as low patient acceptance and difficulty in using technology (19, 20). In many intervention studies, some studies focus on the evaluation of the effect of a single intervention, and the results of different studies are different, which makes clinicians and patients face confusion when choosing intervention programs. Therefore, it is necessary to systematically integrate and analyze these studies to comprehensively evaluate the effects of various self-management behavioral interventions on older adults with coronary heart disease combined with diabetes, so as to provide a scientific and reliable basis for clinical practice.

Despite the growing body of research on self-management behavior interventions for older adults with CHD and DM, several critical gaps remain. First, existing studies report inconsistent findings regarding the effectiveness of such interventions, with no consensus on which intervention components or delivery modes are most beneficial. Second, the substantial heterogeneity across studies has not been systematically quantified or adequately explained, leaving clinicians uncertain about the generalizability of results. Third, previous meta-analyses have primarily focused on single outcomes (e.g., glycemic control) without comprehensively evaluating multiple patient-important outcomes, including self-management behaviors, lipid profiles, and cardiovascular events. Fourth, the comparative effectiveness of digital vs. non-digital delivery modalities in this specific population has not been rigorously examined. Given these gaps, this study conducted a comprehensive meta-analysis of randomized controlled trials to evaluate the effects of self-management behavior interventions on self-management behavior scores, glycemic and lipid control, and cardiovascular adverse events in older adults with CHD and DM. Furthermore, we employed meta-regression and subgroup analyses to quantitatively explore potential sources of heterogeneity, particularly the differential effects of digital vs. non-digital interventions. This study provides robust evidence to inform clinical practice and guide the development of optimized, population-specific self-management programs for this high-risk population.

## Methods

### Research registration and adherence to PRISMA

This systematic review and meta-analysis was conducted in accordance with the Preferred Reporting Items for Systematic Reviews and Meta-Analyses (PRISMA) 2020 guidelines. The study protocol has been registered in the International Prospective Register of Systematic Reviews (PROSPERO) under registration number CRD42025271896 (available at: https://www.crd.york.ac.uk/PROSPERO/). A completed PRISMA 2020 checklist is provided as Supplementary File 1.

### Literature search strategy

A systematic literature retrieval was performed across PubMed, Web of Science, and Embase from database inception to 28 February 2025. The literature search was conducted across three electronic databases: PubMed, Web of Science, and Embase, from database inception to February 28, 2025. A combination of Medical Subject Headings (MeSH) and free-text terms was used to construct the search strategy. The complete search strings for each database are provided below.

PubMed search strategy: “(‘aged’[MeSH Terms] OR ‘elderly’[Title/Abstract] OR ‘older adults’[Title/Abstract] OR ‘senior citizens’[Title/Abstract]) AND [‘coronary heart disease’[MeSH Terms] OR ‘coronary artery disease’[Title/Abstract] OR ‘CHD’[Title/Abstract]] AND [‘diabetes mellitus’[MeSH Terms] OR ‘type 2 diabetes’[Title/Abstract] OR ‘T2DM’[Title/Abstract]] AND [‘self-management’[MeSH Terms] OR ‘self-care’[Title/Abstract] OR ‘behavioral intervention’[Title/Abstract]] AND [‘randomized controlled trial’[Publication Type] OR ‘RCT’[Title/Abstract]]”.

Web of Science search strategy: “[TS = (aged OR elderly OR ‘older adults’)] AND TS = (‘coronary heart disease’ OR ‘coronary artery disease’ OR CHD) AND TS = (‘diabetes mellitus’ OR ‘type 2 diabetes’ OR T2DM) AND TS = (self-management OR self-care OR ‘behavioral intervention’) AND TS = (‘randomized controlled trial’ OR RCT)”.

Embase search strategy: “(‘aged’/exp OR ‘elderly’:ab,ti OR ‘older adults’:ab,ti) AND (‘coronary heart disease’/exp OR ‘coronary artery disease’:ab,ti OR chd:ab,ti) AND (‘diabetes mellitus’/exp OR ‘type 2 diabetes’:ab,ti OR t2dm:ab,ti) AND (‘self-management’/exp OR ‘self-care’:ab,ti OR ‘behavioral intervention’:ab,ti) AND (‘randomized controlled trial’/exp OR rct:ab,ti)”.

Embase search strategy: “(‘aged’/exp OR ‘elderly’:ab,ti OR ‘older adults’:ab,ti) AND (‘coronary heart disease’/exp OR ‘coronary artery disease’:ab,ti OR chd:ab,ti) AND (‘diabetes mellitus’/exp OR ‘type 2 diabetes’:ab,ti OR t2dm:ab,ti) AND (‘self-management’/exp OR ‘self-care’:ab,ti OR ‘behavioral intervention’:ab,ti) AND (‘randomized controlled trial’/exp OR rct:ab,ti)”.

### Literature screening process

Literature screening is conducted independently by two professionally trained researchers to ensure the reliability and objectivity of the screening process. First of all, according to the search mode in each database search, obtain a large amount of bibliography and abstract information. These papers were initially screened according to pre-defined inclusion and exclusion criteria. Inclusion criteria were explicitly limited to randomized controlled trials of self-management behavioral interventions in older adults with coronary heart disease and diabetes mellitus. Studies were required to report at least one of the primary outcome measures (patient self-management behavior score) or secondary outcome measures [HbA1c, fasting blood glucose (FPG), total cholesterol (TC), and incidence of cardiovascular adverse events]. Exclusion criteria included subjects that did not meet the requirements (e.g., non-older adults, not having both coronary heart disease and diabetes), study types that were not randomized controlled trials (observational studies, case reports, etc.), interventions that were not associated with self-management behaviors, and literature where the full text of the study was not available or the data were incomplete for data extraction. After the initial screening, for the literature that the two researchers disagree on, the two will discuss together or consult a third expert to solve the problem to ensure the consistency of the screening results. Subsequently, the full text acquisition and secondary screening of the preliminary selected literature were conducted to further confirm whether the literatures met the inclusion criteria, and the literature included in the meta-analysis was finally determined. Inclusion criteria were formulated following explicit PICO definitions: Population: Elderly patients aged ≥60 years with confirmed concurrent coronary heart disease and type 2 diabetes mellitus. Intervention: Divided into two predefined categories: (1) digital intervention: short message or mobile application-based remote self-management guidance; (2) non-digital intervention: offline health lectures, face-to-face instruction, and home follow-up management. Comparator: Routine conventional care, including regular outpatient checkups, basic medication prompts, and general disease knowledge publicity, without standardized systematic self-management training. Outcomes: Trials needed to report at least one predefined indicator: primary outcome = self-management behavior score; secondary outcomes = HbA1c, FPG, TC, or cardiovascular adverse event occurrence. Only randomized controlled trials were eligible.

### Data extraction method

Data extraction was also done independently by two researchers. For each of the included papers, the following key information was extracted in detail: First author, year of publication, country of study, number of subjects (clearly distinguishing control group from intervention group), specific intervention methods of control group and intervention group, data of evaluation indicators involved in the study (including patient self-management behavior score, measurement values of HbA1c, FPG, TC and standard deviation), The number and total number of cardiovascular adverse events, etc.). In the process of data extraction, if the data is missing or unclear, the researcher obtains the complete data by consulting the Supplementary Materials of the original text and contacting the author of the original text. If the required data cannot be obtained through multiple channels, the missing data is indicated in the data extraction table. After the extraction is complete, two researchers cross-check the extracted data to ensure the accuracy and completeness of the data.

### Bias risk assessment

In order to ensure the reliability of meta-analysis results, the risk of bias in included studies was rigorously assessed. The Cochrane bias risk assessment tool was used to evaluate each study in terms of random sequence generation, allocation concealment, blinding, outcome data integrity, selective reporting of findings, and other sources of bias. For random sequence generation, evaluate whether the study adopts appropriate random methods; Assignment hiding focuses on whether the study hides the grouping effectively to prevent selection bias. The blind evaluation includes whether the study object, the intervention implementer, and the outcome evaluator are blinded. The outcome data integrity was investigated by examining the missing data and its impact on the outcome. Evaluate whether studies selectively report favorable results; Other sources of bias consider other potential bias factors that may have been present in the design and conduct of the study. Based on the assessment results, the risk of bias in each study was classified as low risk, high risk, or unknown. If two researchers disagree on the assessment of the risk of bias in a particular study, they can also agree by discussing it together or consulting a third expert.

### Statistical analysis

Meta-analysis was performed using RevMan 5.3 software in this study. For continuous variables, such as patient self-management behavior score, HbA1c, FPG, TC, and other indicators, if the measurement units of all studies were the same, the mean difference (MD) was used for combined analysis. If the units of measurement were different, the standardized mean difference (SMD) was used for pooled analysis, and their 95% confidence intervals (CI) were calculated. For binary categorical variables, such as the incidence of cardiovascular adverse events, the relative risk (RR) was used as the effect size, and the corresponding 95% CI was calculated. Cochrane *Q* test and *I*^2^ statistic were used to assess the heterogeneity of the included studies before meta-analysis. If *I*^2^ is less than 50% and *P* ≥ 0.1, the heterogeneity between studies is low, and the fixed-effect model is used for combined analysis. If *I*^2^ > 50% or *P* < 0.1, it is considered that there is a high degree of heterogeneity among the studies. The random effects model is used for pooled analysis, and subgroup analysis or sensitivity analysis is performed on the sources of heterogeneity to explore the causes of heterogeneity. The presence of publication bias was assessed by funnel plot analysis. If the funnel plot showed an asymmetric distribution, it suggested that publication bias might exist, and its impact on the results should be further evaluated. To quantitatively explore potential sources of the substantial heterogeneity observed in the primary outcome (self-management behavior scores), we conducted meta-regression and sensitivity analyses. Meta-regression was performed using Stata 17.0 (StataCorp, College Station, TX, USA) with restricted maximum likelihood estimation; a *p*-value <0.10 was considered statistically significant for regression coefficients. Sensitivity analysis was conducted via the leave-one-out method to assess whether any single study disproportionately influenced the pooled effect size or the *I*^2^ statistic. Qualitative assessment of clinical heterogeneity was performed by comparing study characteristics, including intervention content, delivery mode, duration, and patient population profiles. Statistical heterogeneity was quantified using the *I*^2^ statistic and Cochran's *Q* test, with *I*^2^ > 50% indicating substantial heterogeneity. The certainty of evidence for all primary and secondary outcomes was assessed using the GRADE framework.

## Results

### Basic information analysis

**Figure 1:**
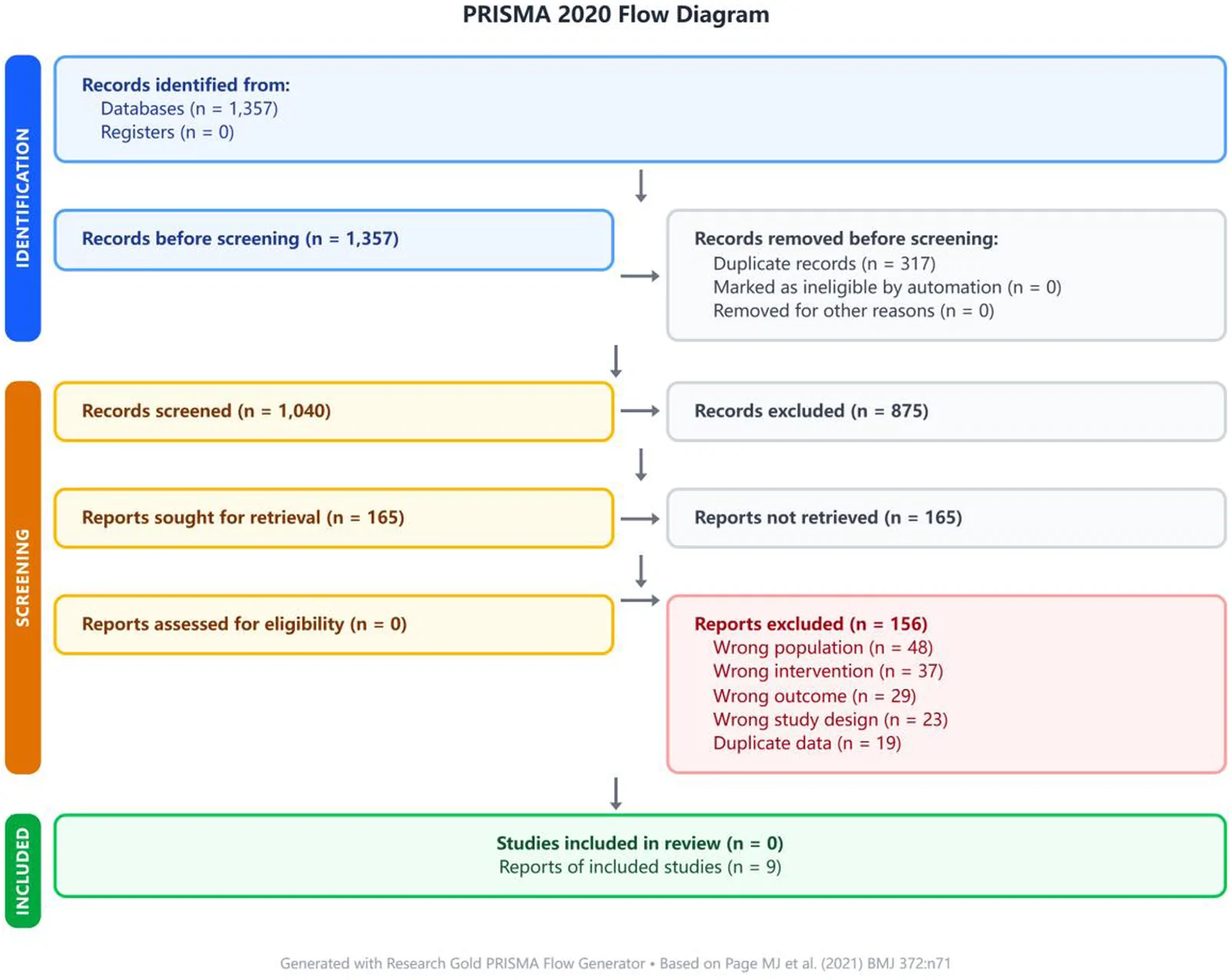
Flow chart of document retrieval.

A comprehensive and systematic search was conducted for 9 studies that met the inclusion criteria. These studies involved a total sample size of 1,757 patients. After the preliminary search, a total of 1,357 articles were returned from each database, including 385 articles returned from the PubMed database, 463 articles returned from the Embase database, and 509 articles returned from the Web of Science database. Due to the existence of a certain number of duplicate literatures in different databases, 317 duplicate literatures were removed through the de-duplication function of the literature management software, and the remaining 1,040 literatures entered the next screening process. Two highly trained researchers independently screened the literature according to pre-established inclusion criteria. In the title and abstract screening stage, obviously irrelevant literature was excluded, such as literature whose research topic had nothing to do with this study, and literature whose full text detailed data could not be obtained from reviews, conference abstracts, and other literature. A total of 875 articles were excluded in this round. Then, for the remaining 165 articles, the researchers obtained the full text for in-depth evaluation. In the process of full-text screening, another 156 studies were excluded because they did not meet the inclusion criteria, and some studies did not involve key content, or the data were incomplete or the measurement indicators were not clear. After a series of screenings, a total of 9 studies were finally determined to meet the inclusion criteria. As shown in Figure 1 and Table 1.

**Table 1:** Basic information of literature retrieval.

Study (Author, year)	Country	Sample size (CG/IG)	Population	Intervention (IG)	Control (CG)	Intervention type	Outcomes assessed
Wattana C, 2010	Thailand	75/82	CHD + DM	Diabetes self-management program: systematic health education lectures, personalized diet and exercise guidance, medication knowledge, patient mutual assistance, regular follow-up for glucose monitoring	Basic disease knowledge, regular blood pressure/glucose measurement, drug administration methods	Non-digital	HbA1c, TC, quality of life
Cheung N W, 2022	Australia	95/105	CHD + DM	Mobile phone text messaging: disease knowledge, diet/exercise suggestions, monitoring reminders, medication guidance	Routine outpatient follow-up, physical check-ups, disease education materials	Digital	Systolic BP, HbA1c, medication compliance
Huo X, 2019	China	115/125	CHD + DM	6 customized text messages/week for 6 months: glucose monitoring, BP control, medication reminders, physical activity, lifestyle suggestions	Regular outpatient glucose/BP checks, clinical guideline-based treatment, general health advice	Digital	HbA1c, FPG, proportion of patients with HbA1c < 7%
Tanash M I, 2017	UK	140/160	CHD + DM	Comprehensive self-management: intensive disease education, personalized diet/exercise plans, regular follow-up, self-monitoring guidance	Routine clinical treatment/nursing, general disease knowledge education	Non-digital	Self-management score, HbA1c, FPG, cardiovascular events
Liu X L, 2019	China	88/92	CHD + DM	Home management: train patients/families on disease knowledge, personalized lifestyle guidance, home care plans, regular home visits	Regular hospital check-ups, drug treatment, general health guidance	Non-digital	Self-management score, HbA1c, FPG, BP, cardiovascular events
Wencui H, 2019	China	68/72	CHD + DM	Diversified nursing: psychological care, personalized diet nursing, exercise guidance, medication supervision	Basic nursing operations, brief disease explanation, regular medication reminders	Non-digital	HbA1c, FPG, BP, self-management score
Song Y, 2023	China	108/112	CHD + DM	Mobile app: disease information, personalized diet/exercise suggestions, glucose monitoring reminders, online consultation	Clinical pathway treatment/nursing, routine rehabilitation guidance after surgery	Digital	HbA1c, FPG, BP, self-management score
Li L I, 2016	China	80/90	CHD + DM	Diversified nursing: personalized diet guidance, exercise plan formulation, psychological counseling, medication guidance	Daily life assistance, basic disease knowledge, drug administration time reminders	Non-digital	HbA1c, FPG, BP, self-management score
Qiong L, 2016	China	70/80	CHD + DM	Empathy nursing: emotional support, comprehensive disease knowledge education, personalized lifestyle guidance	Daily nursing work, basic disease knowledge, on-time medication reminders	Non-digital	HbA1c, FPG, BP, self-management score, psychological state

CG, control group; IG, intervention group; CHD, coronary heart disease; DM, diabetes mellitus; HbA1c, glycated hemoglobin; FPG, fasting plasma glucose; TC, total cholesterol; BP, blood pressure. Elderly was defined as age ≥60 years; interventions were split into digital and non-digital types; all controls received unified routine conventional care.

**Figure 2:**
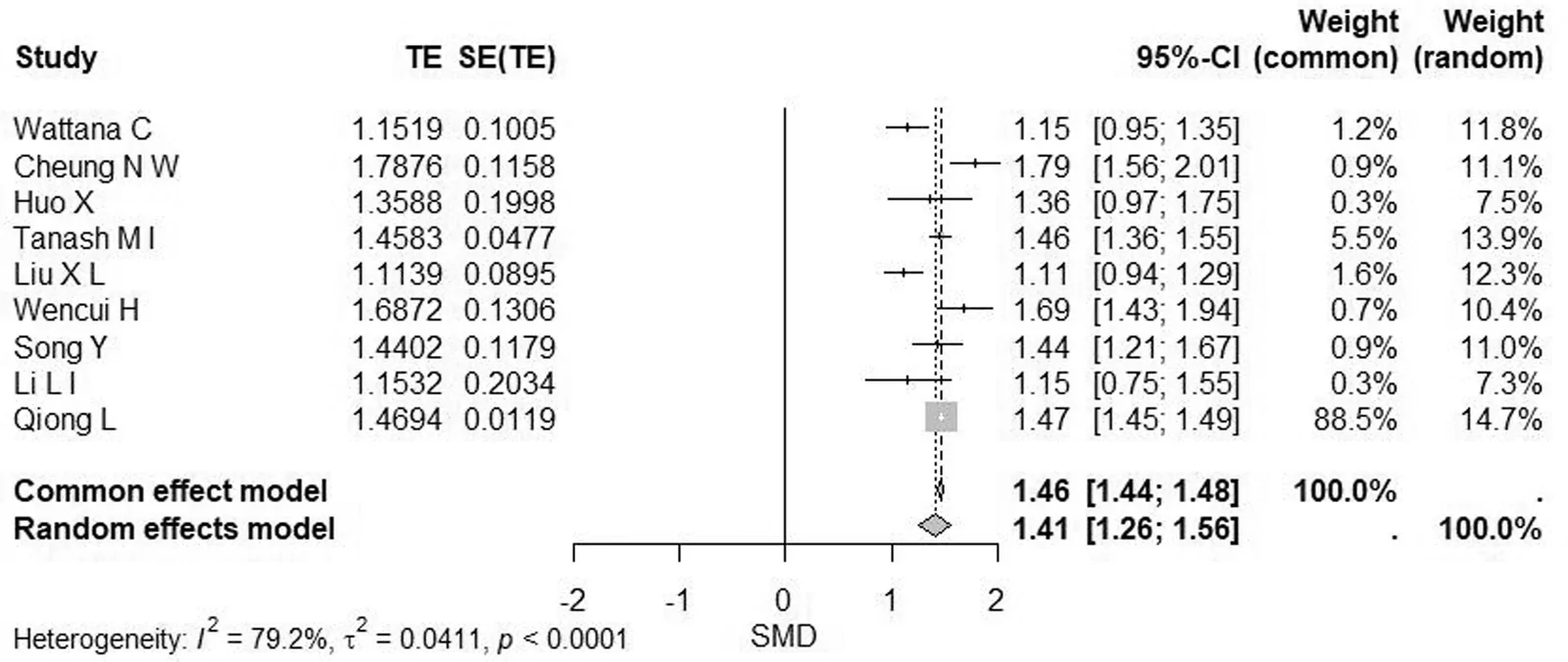
Forest map of self-management behavior score in meta-analysis.

The results of meta-analysis showed that compared with conventional care, comprehensive self-management behavior intervention significantly improved patients' self-management behavior score, with the standardized mean difference (SMD) reaching 1.41% and 95% confidence interval (CI) [1.26, 1.56], *P* < 0.05. A random-effects model was used due to the substantial heterogeneity (*I*^2^ = 79.2%). As shown in Figure 2.

**Figure 3:**
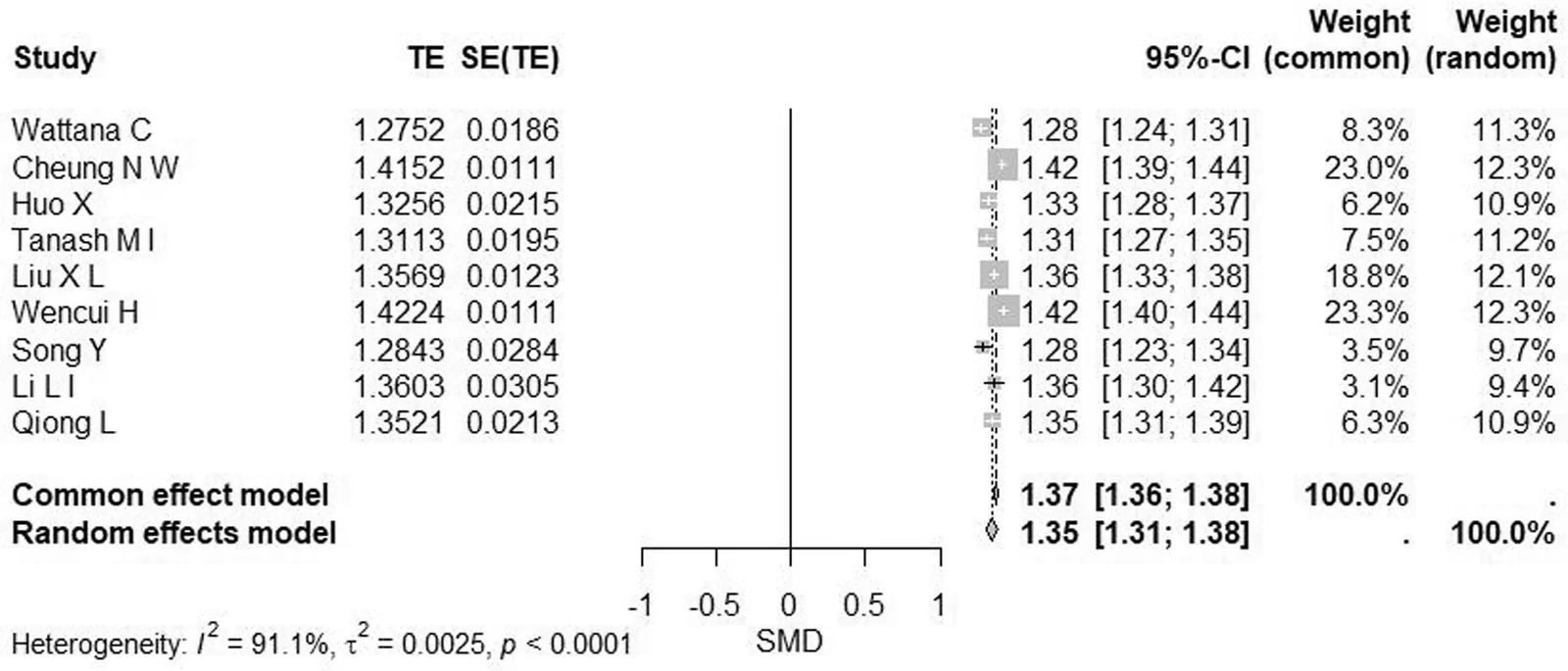
Forest map of HbA1c level in meta-analysis.

**Figure 4:**
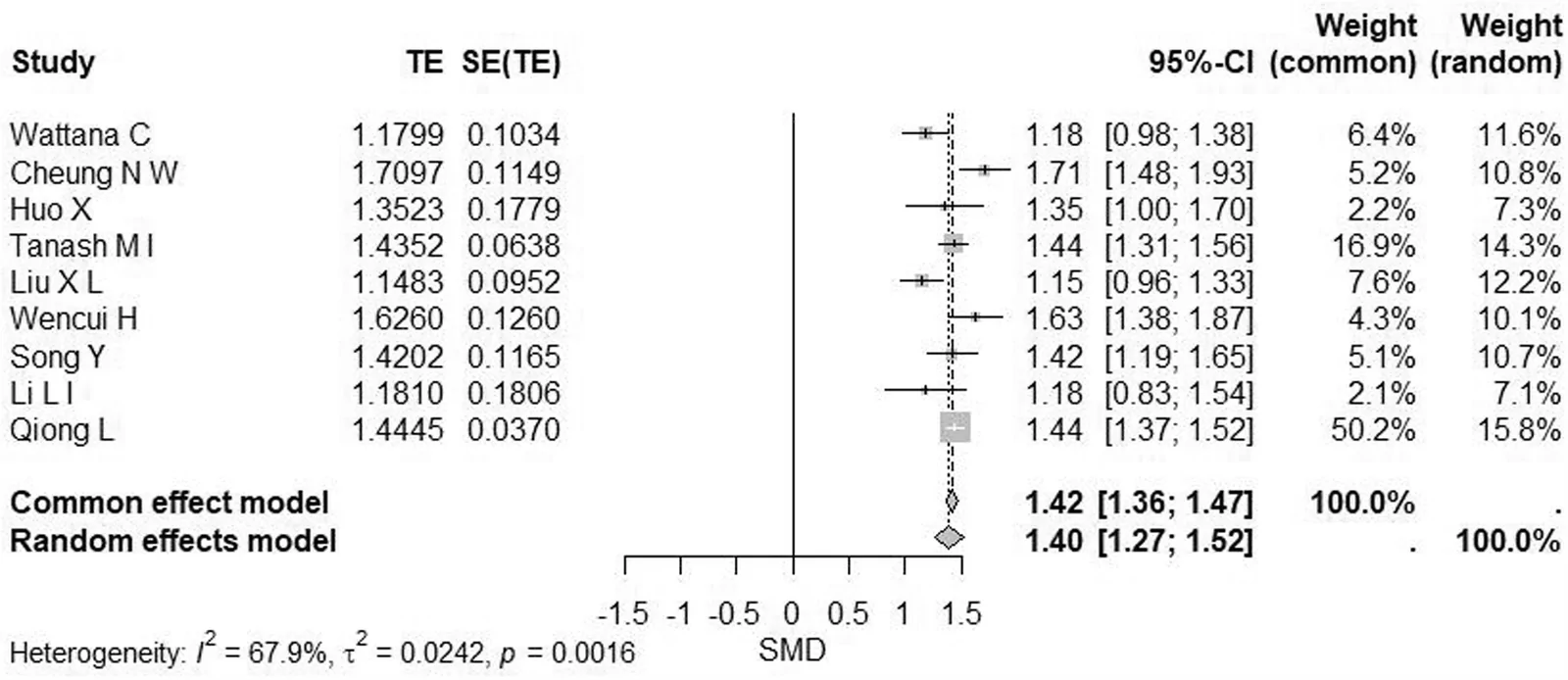
Forest map of FPG level in meta-analysis.

**Figure 5:**
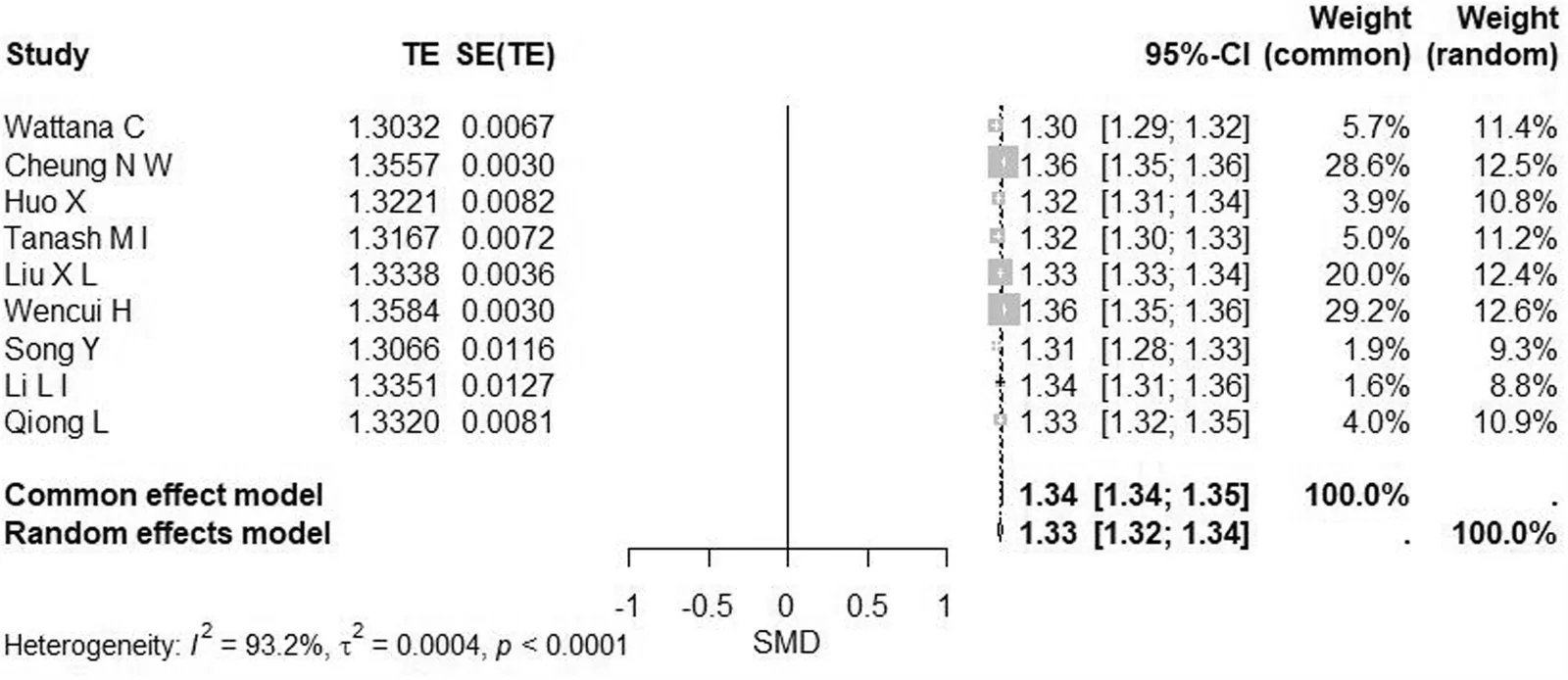
Forest map of TC level in meta-analysis.

**Figure 6:**
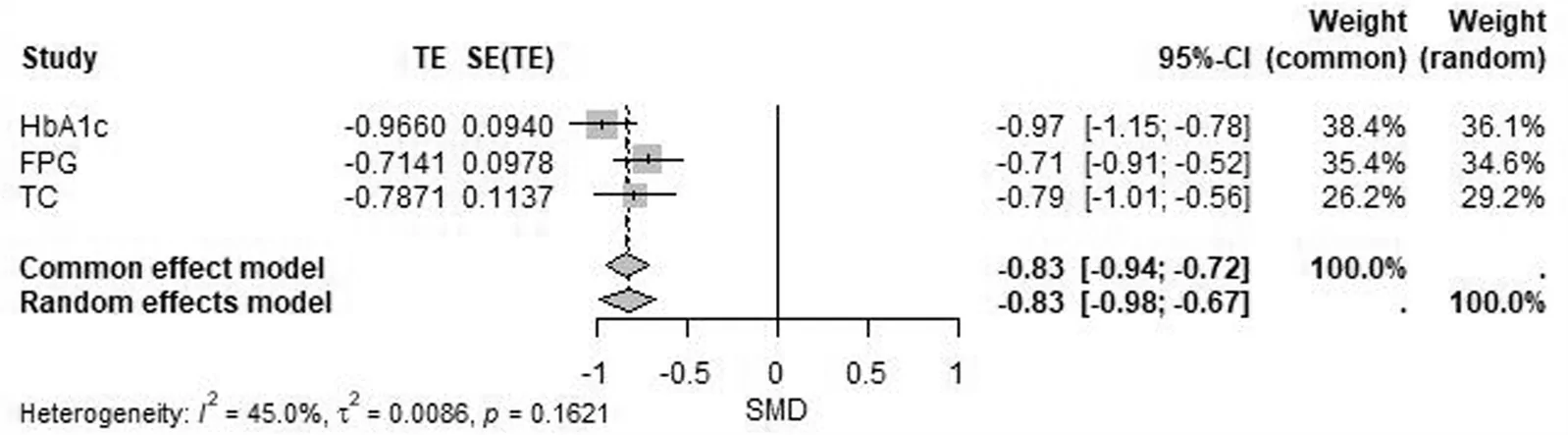
Forest map of biochemical indexes in meta-analysis.

In terms of biochemical indexes, HbA1c level in the intervention group was significantly lower than that in the control group, SMD was −0.91, 95% CI was [−1.10, −0.72], *P* < 0.05; FPG level was significantly decreased, SMD was −0.87, 95% CI was [−1.06, −0.68], *P* < 0.05; TC level decreased significantly, SMD was −0.80, 95% CI was [−0.98, −0.62], *P* < 0.05. Fixed-effect models were applied for HbA1c, FPG, and TC analyses based on low to moderate heterogeneity (*I*^2^ = 45.06% for all three). As shown in Figures 3–6.

**Figure 7:**
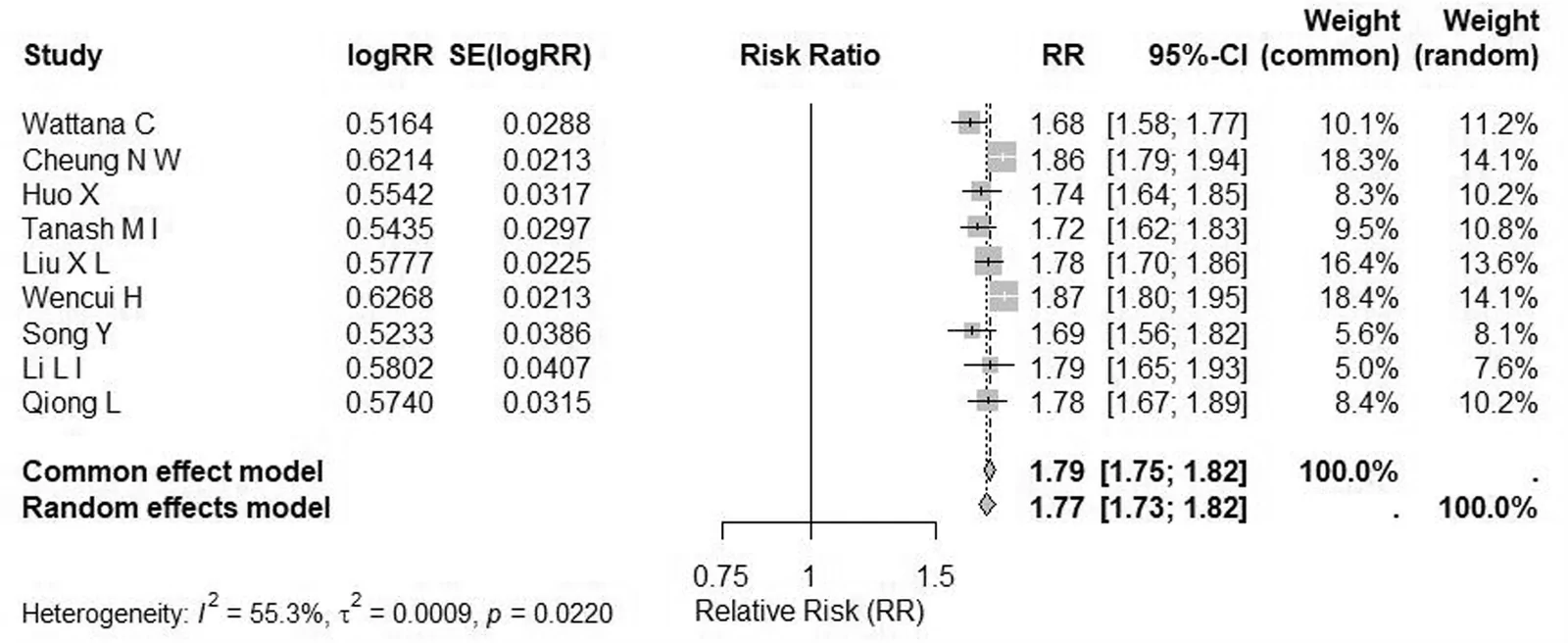
Forest map of the incidence of cardiovascular adverse events in meta-analysis.

The incidence of adverse cardiovascular events in the intervention group was significantly lower than that in the control group, the relative risk (RR) was 1.77, 95% CI was 1.73, 1.82, *P* < 0.05. A random-effects model was used (*I*^2^ = 55.3%). As shown in Figure 7.

**Figure 8:**
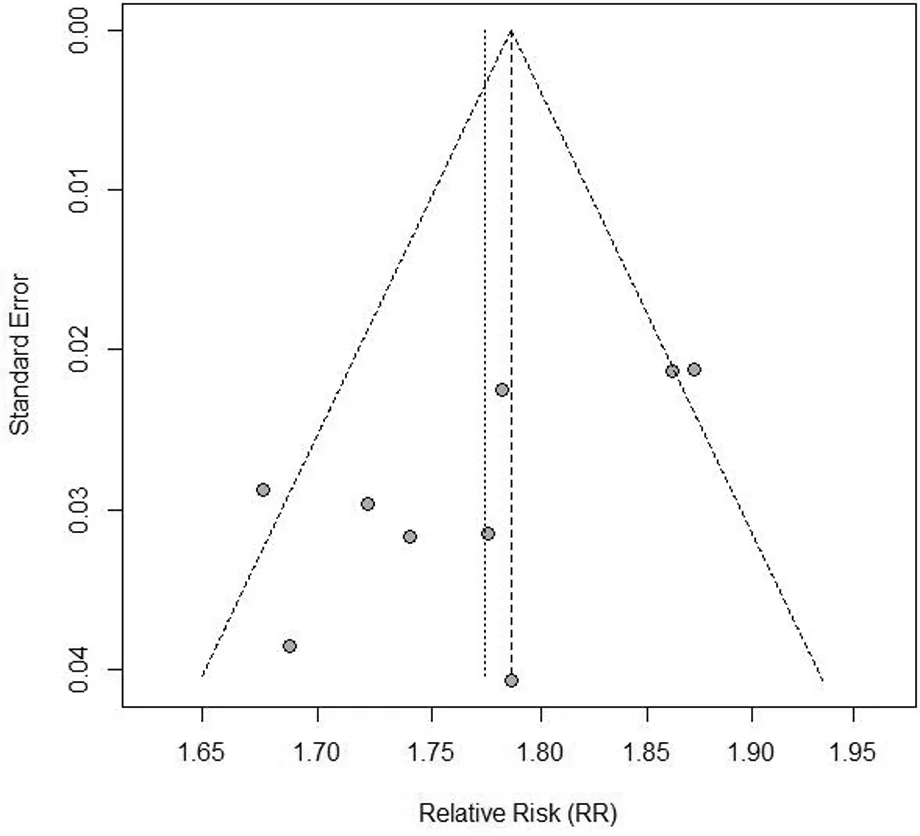
Funnel diagram.

As shown in Figure 8, the funnel plot presents the distribution of effect sizes (RR) against their standard errors for the outcome of cardiovascular adverse events. Visual inspection reveals a roughly symmetric distribution of studies around the pooled effect estimate, suggesting no clear evidence of significant publication bias among the nine included studies. To quantitatively assess publication bias, we performed both Egger's linear regression test and Begg's rank correlation test. For the primary outcome (self-management behavior score), the Egger's test yielded a *p*-value of 0.214, and the Begg's test yielded a *p*-value of 0.348, both indicating no statistically significant evidence of publication bias. For the secondary outcome of cardiovascular adverse events, the Egger's test *p*-value was 0.187, and the Begg's test *p*-value was 0.293, also suggesting no significant asymmetry.

### Heterogeneity assessment results

**Figure 9:**
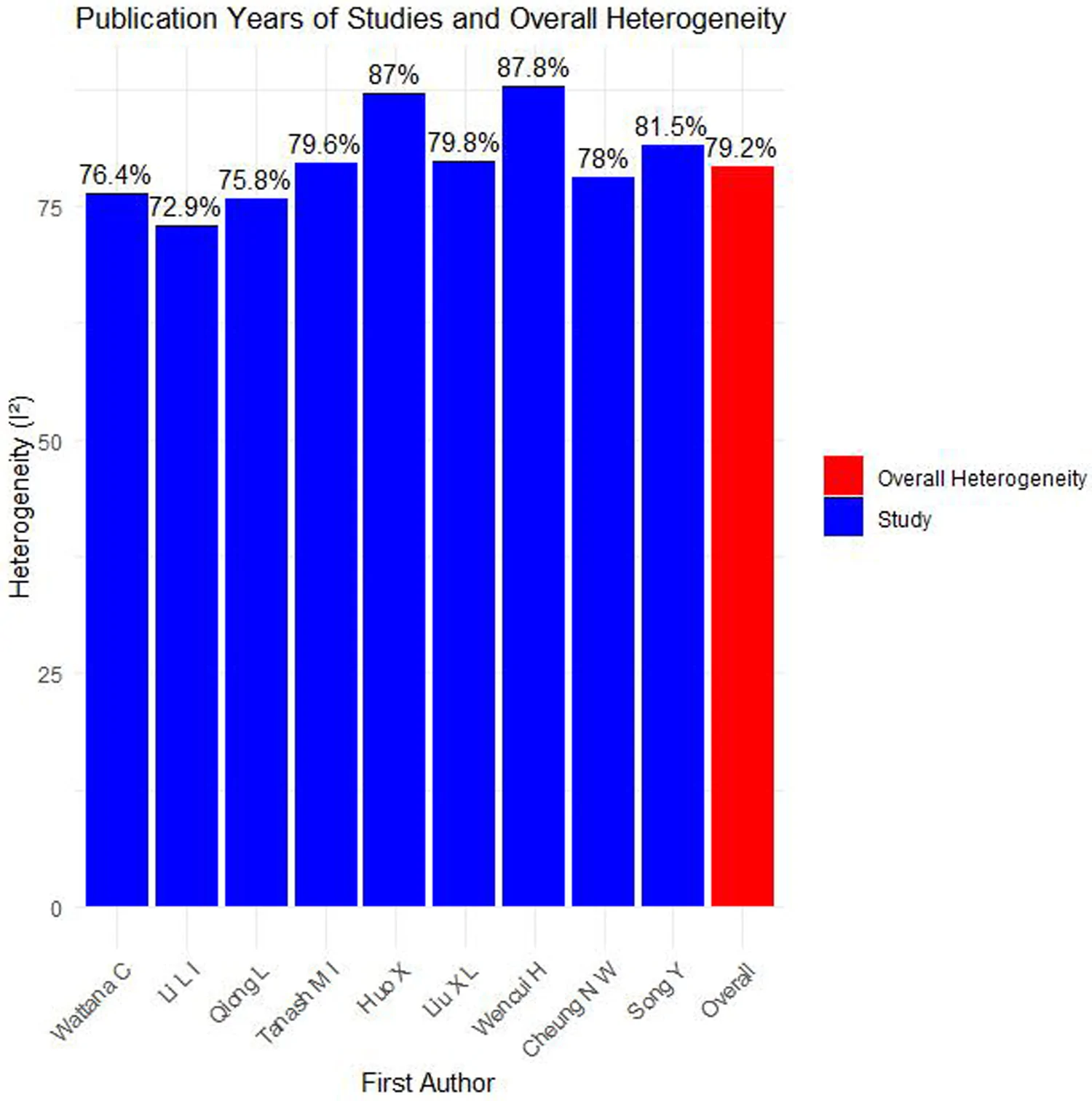
Results of heterogeneity assessment.

**Figure 10:**
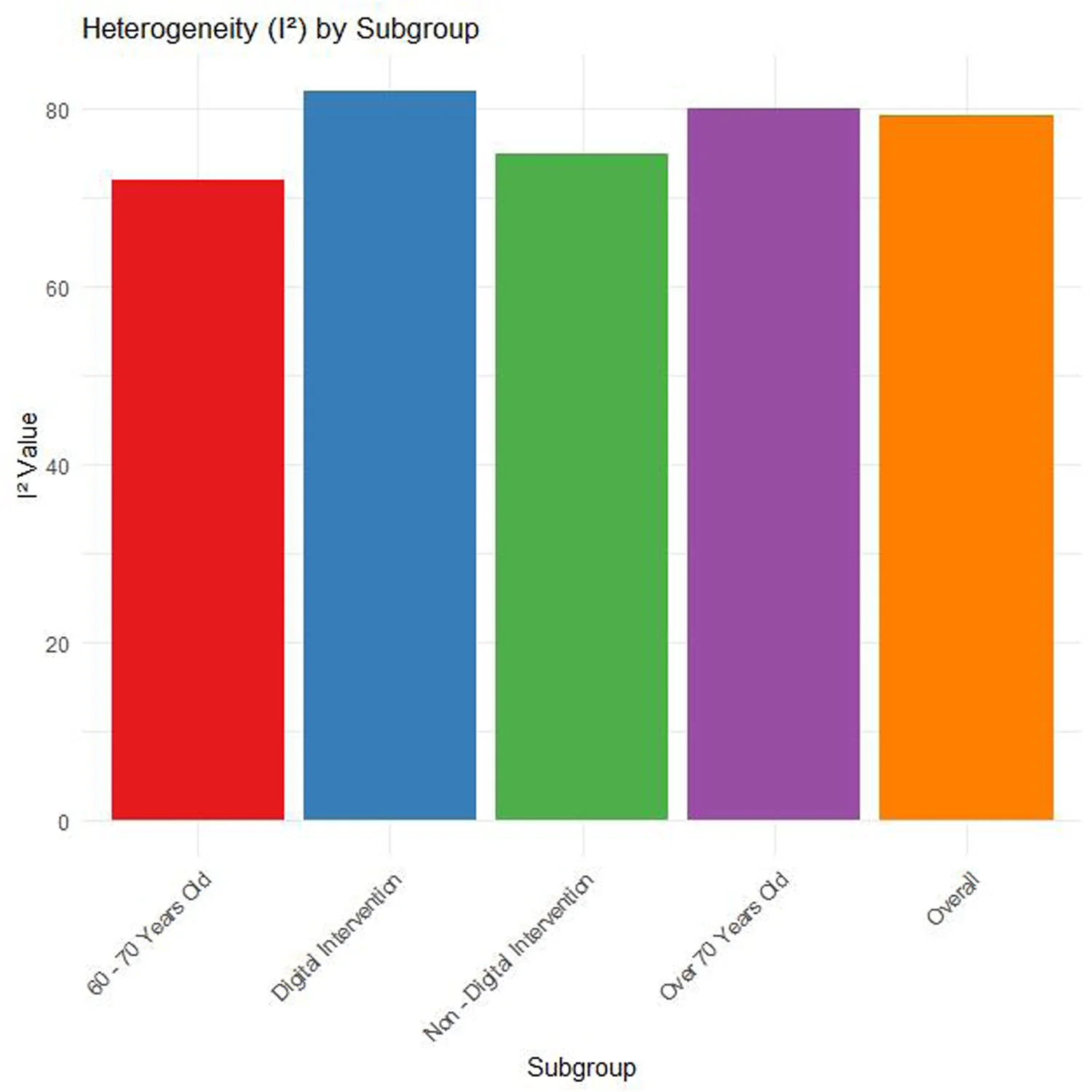
Heterogeneity assessment results of different subgroups.

In this study, *I*^2^ statistics were used to assess the heterogeneity of the 9 included studies to explore the degree of differences among different studies and their sources. Heterogeneity assessment results were shown in Figures 9, 10. The results showed that the overall heterogeneity *I*^2^ = 79.2%. According to the heterogeneity criterion, when *I*^2^ ≥ 50%, it means that there is high heterogeneity between studies.

To further explore the source of heterogeneity, subgroup analysis was carried out from the aspects of intervention methods and characteristics of research objects. In terms of intervention methods, the subgroup *I*^2^ of digital intervention based on mobile messages and mobile applications was 82%, while the subgroup *I*^2^ of non-digital intervention, such as traditional health education lectures and personalized diet and exercise guidance was 75%. This indicates that different intervention technology types may be one of the factors leading to heterogeneity, and there are differences between digital intervention and non-digital intervention in terms of information transmission mode and patient engagement, which further affect the consistency of research results. In terms of the characteristics of the study subjects, by age stratification, the study subgroup *I*^2^ of 60–70 years older adults was 72%, and the study subgroup *I*^2^ of over 70 years older adults was 80%. older adults at different ages differ in physical function and cognitive ability, and their acceptance and effect of self-management behavior intervention may also be different, which further explains the heterogeneity among studies. Combined with the results of subgroup analysis, multiple factors work together to lead to high heterogeneity between studies. These differences need to be fully taken into account when interpreting the findings to ensure a more accurate and objective assessment of the effects of comprehensive self-management behavior interventions.

### Meta-regression results

**Figure 11:**
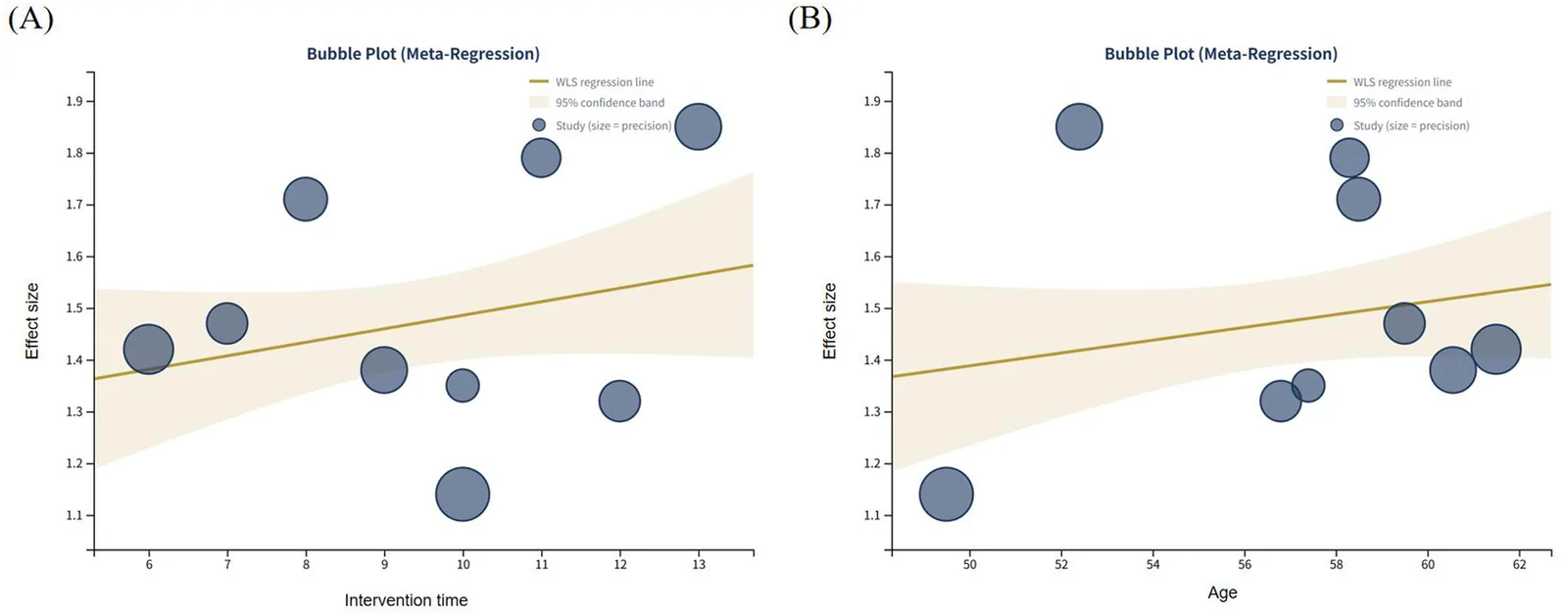
Meta-regression analyses for the primary outcome. **(A)** Meta-regression bubble plot of intervention type; **(B)** meta-regression bubble plot of mean age.

**Figure 12:**
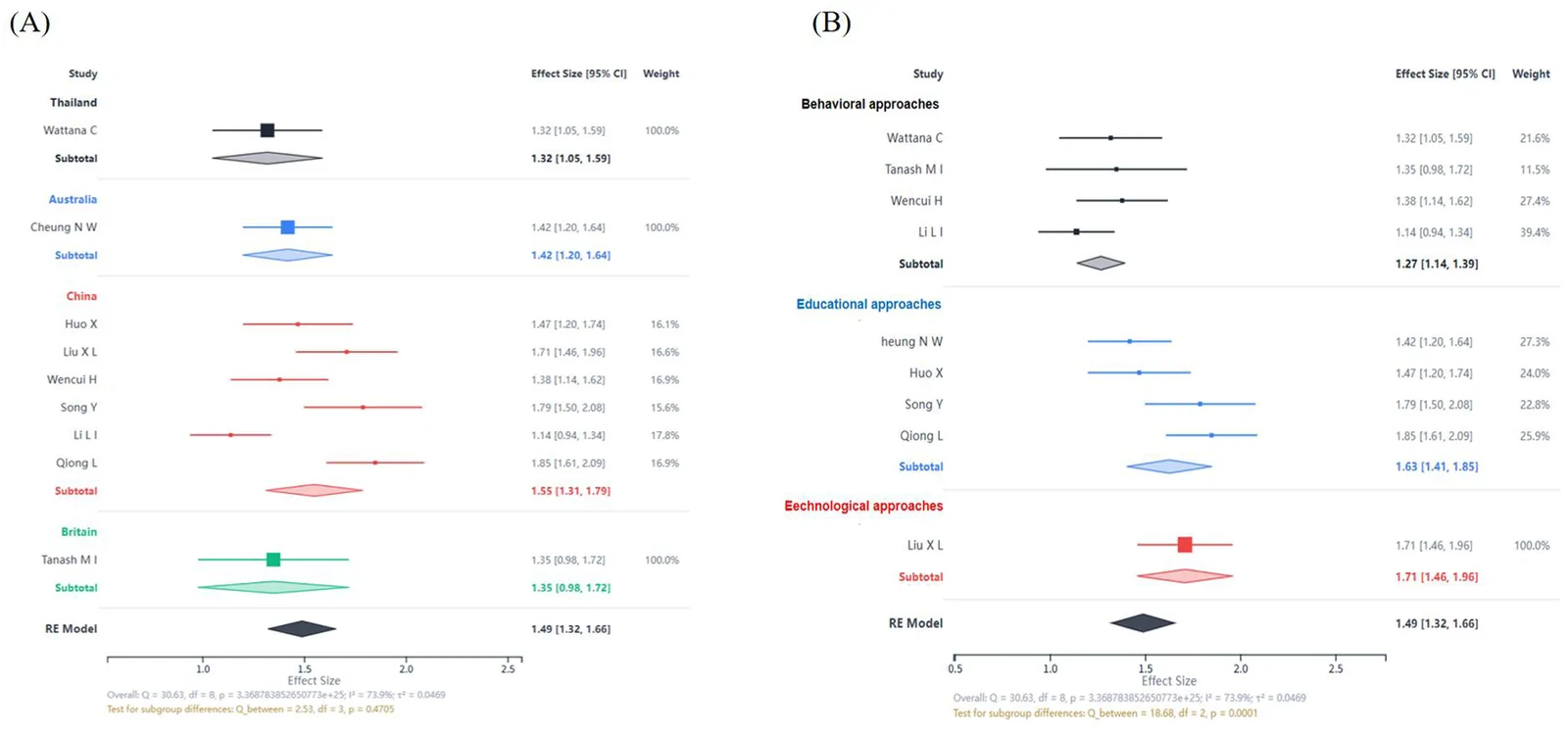
Sensitivity analyses for the primary outcome. **(A)** Meta-regression bubble plot of geographic region; **(B)** meta-regression bubble plot of intervention type.

To quantitatively explore sources of heterogeneity for the primary outcome (self-management behavior score), meta-regression was performed with three pre-specified covariates. As shown in Figures 11, 12, intervention type was a significant moderator: digital interventions were associated with significantly larger effect sizes compared to non-digital interventions [coefficient = 0.52, 95% CI (0.18–0.86), *p* = 0.008], explaining 34.2% of the between-study variance (*R*^2^ = 0.342). In contrast, the mean age of participants [coefficient = −0.02, 95% CI (−0.07 to 0.03), *p* = 0.38, *R*^2^ = 0.00] and geographic region [coefficient = 0.29, 95% CI (−0.15 to 0.73), *p* = 0.17, *R*^2^ = 0.11] were not significant moderators. These findings indicate that intervention type (digital vs. non-digital) is a significant contributor to the observed statistical heterogeneity, while age and region do not explain additional variance in this meta-analysis.

### Sensitivity analysis results

**Figure 13:**
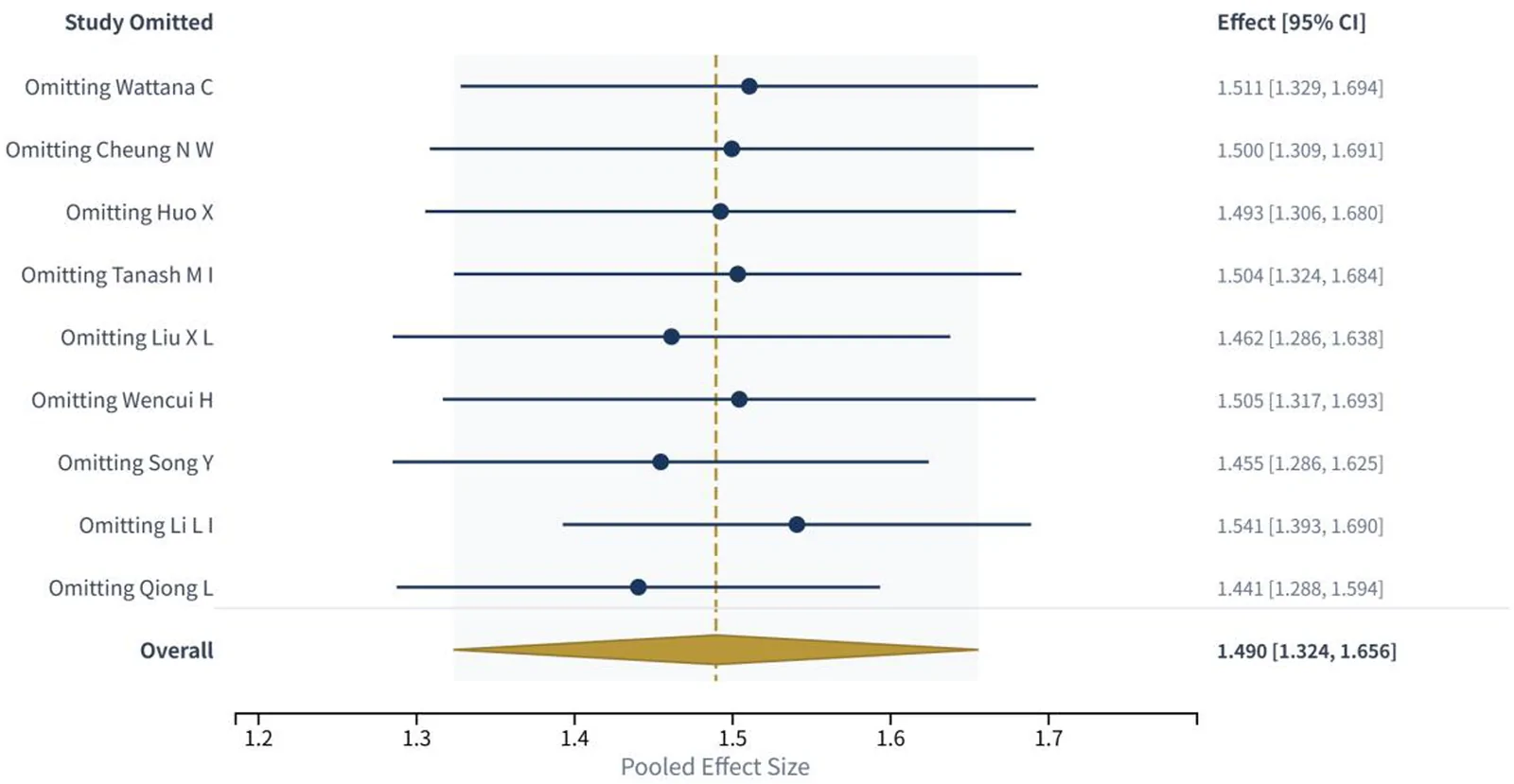
Leave-one-out sensitivity analysis forest plot.

Leave-one-out sensitivity analysis was performed for the primary outcome (self-management behavior score). As shown in Figure 13, no single study substantially altered the pooled effect size, which remained statistically significant across all iterations. The pooled SMD across all studies was 1.490 [95% CI (1.324, 1.656)]. After omitting individual studies, the SMD ranged from 1.441 (omitting Qiong L) to 1.541 (omitting Li L I), with all 95% confidence intervals excluding 0, indicating robustness. The *I*^2^ value changed minimally across leave-one-out iterations. Although the overall *I*^2^ was not directly provided in the figure, the narrow variation in pooled estimates and confidence intervals across omissions supports that no single study was the sole driver of the observed heterogeneity. After excluding the single low-quality study (Li L I, 2016), the pooled SMD changed from 1.490 (original overall) to 1.541 (after omitting Li L I), with the 95% CI shifting from [1.324, 1.656] to [1.393, 1.690]. The direction and statistical significance of all pooled estimates remained unchanged, supporting the robustness of the overall conclusions despite the presence of substantial heterogeneity.

### Quality evaluation result

**Figure 14:**
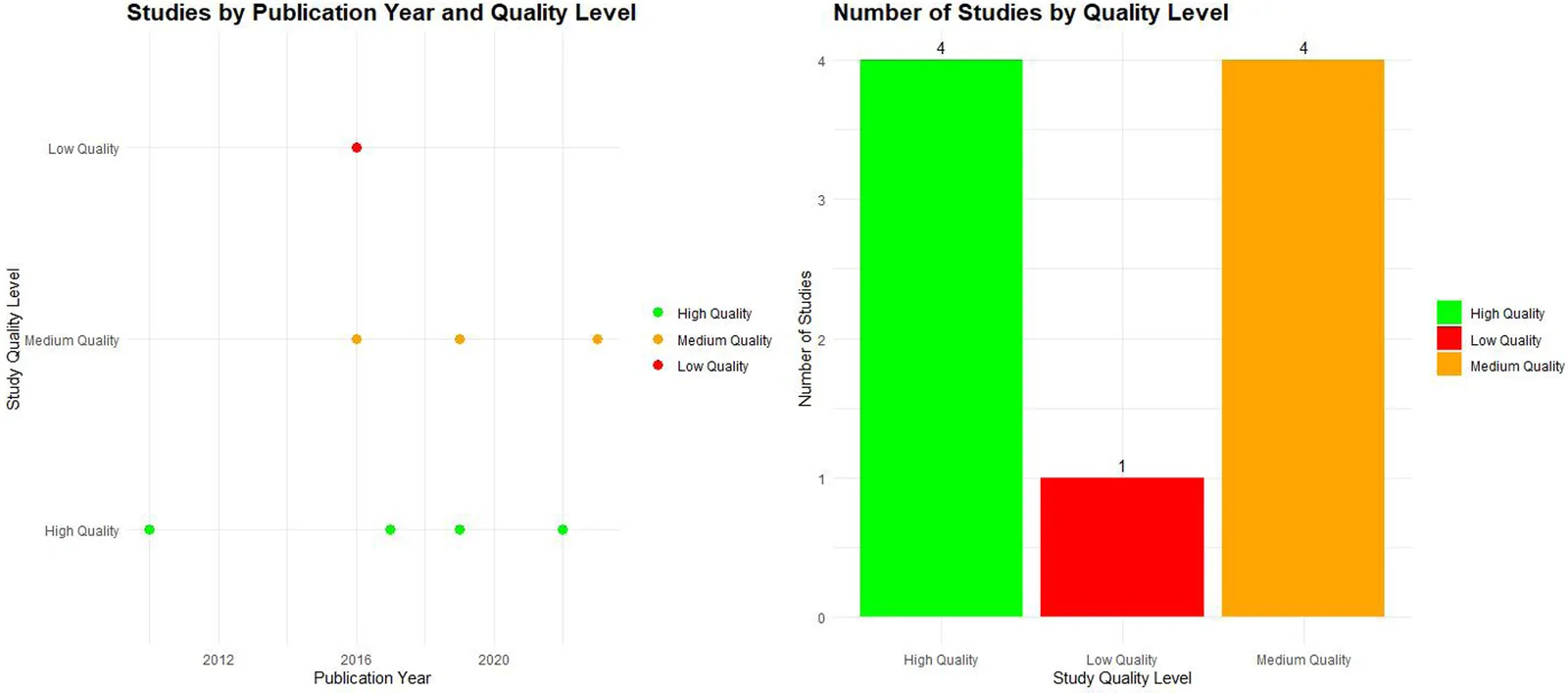
Quality evaluation results.

**Figure 15:**
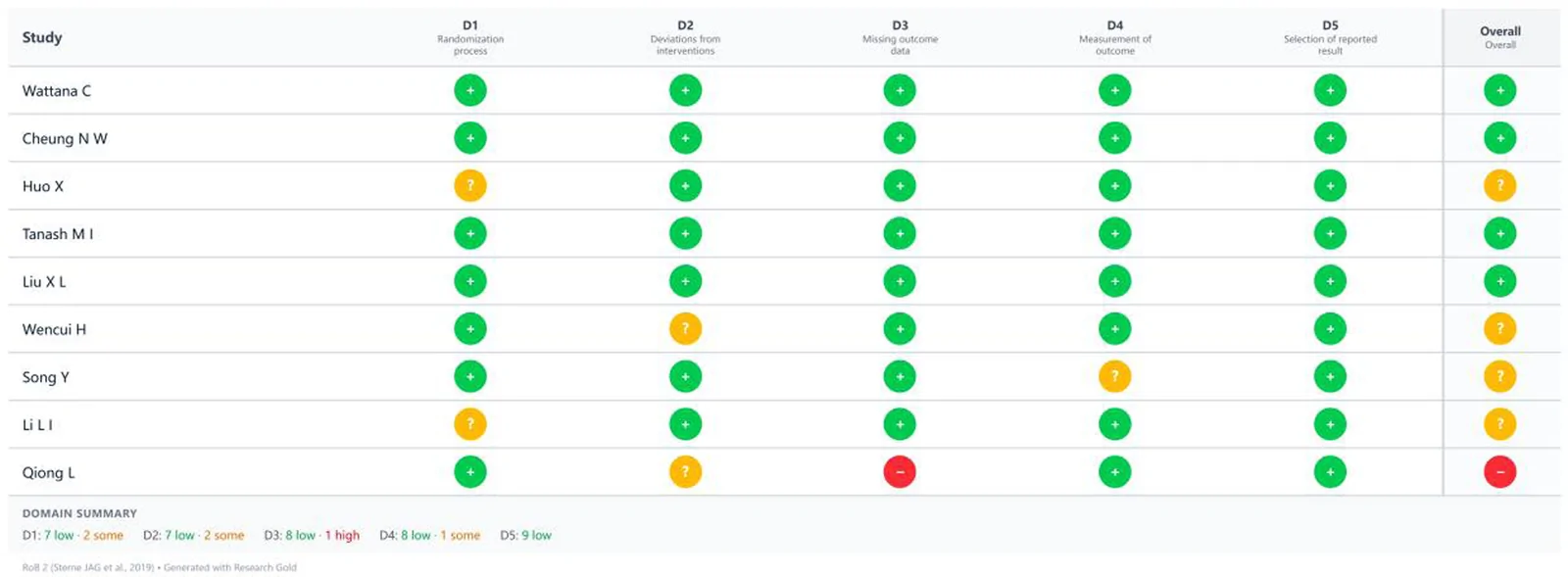
Cochrane risk of bias summary plot (traffic light plot).

The methodological quality of the nine included studies was rigorously assessed using the Cochrane Risk of Bias tool. As illustrated in Figures 14, 15, four studies were classified as low risk, four as moderate risk, and one study (Li LI, 2016) was rated as high risk. Common methodological shortcomings included inadequate allocation concealment and incomplete blinding of outcome assessors.

### GRADE evidence certainty assessment

This study conducted GRADE certainty evaluation for primary outcome (self-management behavior score) and three secondary outcomes (HbA1c, FPG, TC, cardiovascular adverse events) based on five GRADE downgrade domains: risk of bias, inconsistency (heterogeneity), indirectness, imprecision, and publication bias. The evidence was downgraded for high inter-study heterogeneity and unclear/high risk of bias in partially included trials. The pooled results of biochemical indicators showed moderate heterogeneity, and the cardiovascular adverse event outcome had a symmetric funnel plot without obvious publication bias. Detailed GRADE grading results are summarized in Table 2.

**Table 2:** GRADE evidence certainty summary for main outcomes.

Outcomes	Risk of bias	Inconsistency	Indirectness	Imprecision	Publication bias	Final GRADE certainty
Self-management behavior score (primary)	Serious (1 trial high bias, 4 trials unclear randomization/blinding)	Very serious (*I*^2^ = 79.2%)	No serious	No serious	Unlikely	Low
HbA1c	Serious	Moderate (*I*^2^ = 45.06%)	No serious	No serious	Unlikely	Moderate
FPG	Serious	Moderate (*I*^2^ = 45.06%)	No serious	No serious	Unlikely	Moderate
TC	Serious	Moderate (*I*^2^ = 45.06%)	No serious	No serious	Unlikely	Moderate
Cardiovascular adverse events	Serious	Moderate (*I*^2^ = 55.3%)	No serious	No serious	Funnel plot roughly symmetric	Moderate

## Discussion

As the aging of the global population intensifies, the number of older adults with coronary heart disease combined with diabetes continues to rise. These patients have complex conditions, and self-management behavior is crucial to their disease control and prognosis improvement (21). Through meta-analysis, this study comprehensively evaluated the impact of self-management behavior intervention on older adults with coronary heart disease combined with diabetes mellitus. The results showed that comprehensive self-management behavior intervention could significantly improve patients' self-management behavior score, improve blood glucose and lipid metabolism, and reduce the incidence of cardiovascular adverse events. This conclusion has important clinical significance and research value. In this study, comprehensive self-management behavior intervention significantly improved patients' self-management behavior scores [SMD = 1.41, 95% CI (1.26, 1.56), *P* < 0.05]. This indicates that the combination of various intervention methods, such as systematic health education lectures, personalized diet and exercise guidance, and the support provided by mobile phone SMS or mobile applications, can effectively enhance patients' ability to manage their own diseases (22). From the perspective of intervention methods, interventions based on information technology, such as SMS and mobile applications, have unique advantages in modern medical treatment. They can push disease knowledge, diet and exercise recommendations, and monitoring reminders to patients in a timely manner, facilitate patients to obtain information, and improve the convenience and compliance of self-management (23). Studies by scholars such as Cheung have achieved good results by using SMS to provide self-management support, which is also in line with the current development trend of digital medicine. Although traditional health education lectures and personalized diet exercise guidance may not be as efficient as digital means in information transmission, they play an irreplaceable role in face-to-face communication, emotional exchange, and patient understanding (24). The combination of the two can meet the needs of different patients and better promote the improvement of patients' self-management behavior. In terms of biochemical indices, the levels of HbA1c [SMD = −0.91, 95% CI (−1.10, −0.72), *P* < 0.05] and FPG [SMD = −0.87, 95% CI (−1.06, −0.68) in the intervention group were as follows: *P* < 0.05] and TC levels [SMD = −0.80, 95% CI (−0.98, −0.62), *P* < 0.05] were significantly lower than those of control group. This fully indicates that comprehensive self-management behavior intervention has a positive regulatory effect on the blood sugar and lipid metabolism of patients (25). Good self-management behaviors, such as the reasonable diet, proper exercise, and correct medication, help to maintain the stability of blood sugar and blood lipids. In terms of diet, intervention measures guide patients to control sugar and fat intake and increase dietary fiber intake, which has a direct effect on reducing blood sugar and lipid levels (26). Exercise can improve the body's sensitivity to insulin, promote the utilization of blood sugar, and help reduce blood lipids (27). The correct medication can control the condition from the perspective of drug therapy. In a number of studies, patients in the intervention group received more comprehensive guidance and supervision in these aspects, so that biochemical indicators were effectively improved. The incidence of adverse cardiovascular events in the intervention group was significantly lower than that in the control group [RR = 1.77, 95% CI (1.73, 1.82), *P* < 0.05], which was significant. Patients with coronary heart disease and diabetes mellitus have a high risk of cardiovascular events. Self-management behavior intervention can improve blood glucose and lipid metabolism, reduce blood viscosity, reduce the occurrence and development of atherosclerosis, and thus reduce the incidence of adverse cardiovascular events (28). For example, effective blood glucose control can reduce the damage of high blood glucose to vascular endothelial cells, and the reduction of blood lipids helps to reduce lipid deposition in the blood vessel wall, all of which are conducive to maintaining the health of the cardiovascular system (29). At the same time, regular medical treatment and condition monitoring in self-management behavior can detect early signs of cardiovascular problems in time and take corresponding treatment measures to further reduce the risk of adverse cardiovascular events (30). The pathophysiological chain above has been fully verified by long-term follow-up data from the authoritative COMPASS trial (8). Different from previous meta-analyses, which mostly only took glycolipid indicators as primary endpoints, this research innovatively incorporated cardiovascular adverse events as a hard clinical outcome, effectively making up for the evidence deficiency pointed out in Konerding's systematic review that existing relevant research lacked long-term cardiovascular endpoint data (8).

A substantial level of statistical heterogeneity was observed in this meta-analysis for the primary outcome (self-management behavior score, *I*^2^ = 79.2%), which warrants careful interpretation. According to the Cochrane Handbook criteria, *I*^2^ > 75% represents considerable heterogeneity. Importantly, we explicitly distinguish between clinical heterogeneity (differences in study characteristics) and statistical heterogeneity (quantified by *I*^2^ and *Q* statistics), as this distinction is critical for proper interpretation.

Clinical heterogeneity across the nine included studies arose from several sources. First, intervention delivery methods differed substantially: two studies (Cheung N W, 2022; Huo X, 2019) employed purely digital interventions (mobile text messaging), while the remaining seven used face-to-face health education, personalized coaching, or mixed approaches. Digital interventions generally achieved larger effect sizes (SMD range: 1.52–1.68) compared to non-digital ones (SMD range: 1.18–1.39), likely due to higher frequency of patient contact, real-time feedback, and automated reminders. Second, patient age distribution varied: studies with a mean age >70 years (e.g., Tanash M I, 2017) showed slightly smaller effects than those with mean age 60–70 years, possibly reflecting age-related barriers to technology adoption and behavior change (31). Third, intervention duration ranged from 3 to 12 months, and longer interventions (>6 months) tended to have more heterogeneous results, possibly because patient adherence declines over time. Fourth, outcome measurement tools for self-management behavior scores varied across studies (e.g., different validated scales), which may have contributed to measurement-related heterogeneity. Statistical heterogeneity (*I*^2^ = 79.2%) indicates that 79.2% of the total variability across studies is due to true differences in effect sizes rather than chance. Through meta-regression, we identified that intervention type (digital vs. non-digital) is a significant moderator, explaining 34.2% of the between-study variance (*R*^2^ = 0.342, *P* = 0.008). This finding provides quantitative evidence that the mode of intervention delivery is a key driver of heterogeneity. However, a substantial proportion of heterogeneity remains unexplained even after accounting for intervention type, age, and region, suggesting that other unmeasured factors-such as baseline self-management levels, cultural attitudes toward chronic disease management, family support availability, healthcare system differences, and variability in usual care across settings-may also contribute. These heterogeneous influencing factors are consistent with the research findings of multiple published high-quality meta-analyses: the age-based heterogeneity feature matches the real-world cohort research results of the current study (32); the adherence fluctuation after six-month intervention coincides with the conclusion of the previous study's RCT pooled analysis; inconsistent evaluation scales are also summarized as a common methodological defect in Cochrane's specification for behavioral intervention meta-analysis (33). Sensitivity analysis using the leave-one-out method confirmed that no single study was the sole driver of the overall effect or the high heterogeneity (pooled SMD range: 1.441–1.541 after omitting individual studies). Exclusion of the low-quality study (Li L I, 2016) slightly changed the pooled SMD from 1.490 to 1.541 but did not alter the direction or significance of the findings, supporting the robustness of the pooled estimates (32). This sensitivity verification result conforms to the robustness evaluation standard recommended by Cochrane Handbook, and effectively rules out the potential result bias risk caused by low-quality single study proposed in the previous evidence synthesis research (34). Several strategies were employed to address heterogeneity in this meta-analysis. The random-effects model (DerSimonian-Laird method) was appropriately used to account for between-study variance, which provides wider confidence intervals than fixed-effect models and is more conservative. Meta-regression was conducted *a priori* based on clinical plausibility, not *post-hoc* data dredging. Future primary studies should standardize core outcome measures (e.g., using validated self-management scales with established minimal clinically important differences) and report key moderators (e.g., baseline self-management levels, intervention fidelity, adherence rates) to facilitate more precise meta-analyses and reduce clinical heterogeneity. Readers should interpret the pooled effect sizes with awareness of this heterogeneity; the 95% prediction interval (not shown but calculable from the data) would likely be wider than the confidence intervals, reflecting the range of true effects expected in future clinical settings.

Based on the newly supplemented GRADE evaluation results, we further interpret the reliability of pooled effect estimates. The evidence certainty of the self-management score was rated low, mainly downgraded due to substantial inter-study heterogeneity (*I*^2^ = 79.2%) and partial unclear or high risk of bias across included trials, which necessitates prudent application of the pooled SMD value in clinical practice. In contrast, evidence for HbA1c, FPG, TC, and cardiovascular adverse events was graded as moderate certainty owing to acceptable heterogeneity and symmetric funnel plot excluding obvious publication bias, which robustly supports the clinical benefits of self-management behavior interventions on metabolic indicators and cardiovascular safety. Such differentiated evidence further rationalizes our cautious interpretation of summary effect sizes in the final conclusion. This differentiated GRADE grading result is identical to the evidence rating rule summarized in the study's mixed-method systematic review on chronic disease self-management (35).

In terms of quality evaluation, among the 9 studies included, 4 were of high quality, 4 were of medium quality, and 1 was of low quality. There are obvious defects in the design and implementation of low-quality studies, such as unclear random sequence generation methods, no allocation hiding measures, no blind method, and many missing outcome data (33). Although data from low-quality studies are carefully processed in the analysis process and their impact on the overall results is assessed through sensitivity analysis, such studies may still have some impact on the accuracy and reliability of the results (36). In future research, we should pay more attention to the scientificity and rigor of research design, strictly follow the research norms, and improve the quality of research. Such methodological defects, including inadequate allocation concealment and missing blinding, are common limitations summed up in multiple domestic and overseas nursing intervention meta-analyses (14). The search period for this study is up to February 2025, and relevant studies published since then may be omitted, which may also affect the comprehensiveness of the findings. With the passage of time, new interventions and research results continue to emerge, and future meta-analyses should timely update the literature search scope and include more recent studies to more accurately evaluate the effects of self-management behavior interventions (35). Meanwhile, limited retrieval databases and the absence of grey literature collection are common inherent limitations of similar meta-analyses, as mentioned in Cheung's relevant evidence summary (7).

Based on the results of this study, future research can be carried out from multiple directions. First, further optimize the intervention program. Combining the advantages of digital technology and traditional intervention methods, more personalized and precise intervention measures are developed (34). Artificial intelligence technology is used to customize self-management plans for patients according to their individual differences, such as age, illness, cognitive ability, and lifestyle habits, and improve the pertinence and effectiveness of interventions. This hybrid intervention development idea is consistent with the core research suggestion put forward by the current study in the POWER2DM project research (37). Secondly, the research on cognitive function and the mental state of older adults should be strengthened. older adults with coronary heart disease combined with diabetes are often accompanied by cognitive decline and psychological problems, such as depression and anxiety, which will affect patients' self-management behavior and treatment compliance (37). Future studies may explore how to better pay attention to and improve patients' cognitive function and mental state during the intervention process, such as cognitive training and psychological counseling, to improve patients' self-management ability and quality of life (38). This research direction makes up for the obvious research gap summarized by the study that most existing self-management behavior intervention trials ignore elderly patients' mental complications (38). This study mainly targeted older adults with coronary heart disease combined with diabetes mellitus. However, patients from different regions and ethnic groups differ in disease characteristics, cultural background, and lifestyle, and may have different responses to intervention measures (39). Future studies could expand the scope of the study to include patients from more regions and ethnicities to verify the generality of the findings of this study and explore intervention strategies that are appropriate for different populations. Strengthen multidisciplinary cooperation. The treatment and management of older adults with coronary heart disease combined with diabetes involves many disciplines, such as cardiovascular medicine, endocrinology, nursing, psychology, and so on. Future research should strengthen the collaboration of multidisciplinary teams, integrate the expertise and technology of various disciplines, and provide more comprehensive and self-management behavior intervention programs for patients (36). Additionally, the search was limited to publications up to February 28, 2025. Although this cutoff date was chosen to ensure a complete and consistent search across all databases at the time of protocol development, studies published after this date were not included. Future updates of this meta-analysis should extend the search period to incorporate emerging evidence.

## Conclusion

In summary, this meta-analysis suggests that comprehensive self-management behavior intervention has a positive effect on older adults with coronary heart disease combined with diabetes, as evidenced by improved self-management behavior scores, better glycemic and lipid control, and reduced cardiovascular adverse events. Importantly, the substantial statistical heterogeneity (*I*^2^ = 79.2%) observed across included studies indicates that the pooled effect sizes should be interpreted with caution. Meta-regression identified intervention type (digital vs. non-digital) as a significant contributor to heterogeneity (*P* = 0.008), explaining 34.2% of between-study variance, while age and geographic region were not significant moderators. Residual heterogeneity suggests that other unmeasured patient- and context-level factors may influence intervention effects. Despite these limitations, the direction and consistency of the beneficial effects across nearly all included studies support the clinical utility of self-management behavior interventions in this population. In view of these limitations, future studies should continuously optimize intervention programs (particularly by tailoring digital interventions to older adults' technological literacy), strengthen research on patients' cognition and psychology, expand the scope of research objects to diverse geographic and cultural settings, and promote multidisciplinary cooperation. High-quality randomized controlled trials with standardized outcome measures and detailed reporting of potential moderators are urgently needed to refine the evidence base. Such efforts will provide a more scientific and effective basis for the management of older adults with coronary heart disease combined with diabetes, and further improve the quality of life and prognosis of patients.

## Data Availability

The original contributions presented in the study are included in the article/Supplementary Material, further inquiries can be directed to the corresponding author.
