# Supplementary material for: Meta-analysis of the effect of self-management behavior intervention measures in older adults with coronary heart disease and diabetes mellitus
Source: Front Cardiovasc Med. 2026 Aug 12;13:1821784. doi: 10.3389/fcvm.2026.1821784 (PMC13506895; doi:10.3389/fcvm.2026.1821784)
Supplement: Supplementary file 1 [file Table1.docx]

## PRISMA 2020 Checklist

PRISMA 2020 For Systematic Reviews and Meta-Analyses Checklist

| Section and Topic | Item # | Checklist Item | Location where item is reported |
| --- | --- | --- | --- |
| TITLE |  |  |  |
| Title | 1 | Identify the report as a systematic review, meta-analysis, or both. | Title page |
| ABSTRACT |  |  |  |
| Abstract | 2 | See PRISMA 2020 for Abstracts checklist. | Abstract |
| INTRODUCTION |  |  |  |
| Rationale | 3 | Describe the rationale for the review in the context of existing knowledge. | Introduction |
| Objectives | 4 | Provide an explicit statement of the objective(s) or question(s) the review addresses. | Introduction |
| METHODS |  |  |  |
| Eligibility criteria | 5 | Specify the inclusion and exclusion criteria for the review. | Methods section 2.3 |
| Information sources | 6 | Specify all databases, registers, websites, organisations, and other sources searched. | Methods section 2.2 |
| Search strategy | 7 | Present the full search strategies for all databases, registers and websites. | Methods section 2.2 |
| Selection process | 8 | Specify the methods used to decide whether a study met the inclusion criteria. | Methods section 2.3 |
| Data collection process | 9 | Describe the methods used to collect data from reports. | Methods section 2.4 |
| Data items | 10 | List and define all outcomes for which data were sought. | Methods section 2.4 |
| Study risk of bias assessment | 11 | Specify the methods used to assess risk of bias in the included studies. | Methods section 2.5 |
| Effect measures | 12 | Specify for each outcome the effect measure(s). | Methods section 2.6 |
| Synthesis methods | 13 | Describe the methods used to synthesise results. | Methods section 2.6 |
| Reporting bias assessment | 14 | Describe the methods used to assess possible bias in the synthesis. | Methods section 2.6 |
| Certainty assessment | 15 | Describe the methods used to assess certainty (or confidence) in the body of evidence. | Methods section 2.5-2.6 |
| RESULTS |  |  |  |
| Study selection | 16 | Describe the results of the search and selection process. | Results section 3.1 |
| Study characteristics | 17 | Cite each included study and present its characteristics. | Table 1 |
| Risk of bias in studies | 18 | Present assessments of risk of bias for each included study. | Results section 3.5, Figure 11 |
| Results of individual studies | 19 | For all outcomes, present the results for each study. | Results section 3.1, Table 1 |
| Results of syntheses | 20 | Present the results of syntheses and meta-analyses. | Results section 3.1-3.3, Figures 2-7 |
| Reporting biases | 21 | Present assessments of reporting biases. | Results section 3.4, Figure 8 |
| Certainty of evidence | 22 | Present assessments of certainty (or confidence) in the body of evidence. | Results section 3.4, Figure 9-10 |
| DISCUSSION |  |  |  |
| Discussion | 23 | Provide a general interpretation of the results. | Discussion |
| OTHER INFORMATION |  |  |  |
| Registration and protocol | 24 | Provide registration information and where the protocol can be accessed. | Methods section 2.1 |
| Support | 25 | Describe sources of financial or non-financial support. | Funding section |
| Competing interests | 26 | Declare any competing interests of review authors. | Conflict of interest section |
| Availability of data | 27 | Report whether the study data will be made available. |  |
